# Oxidative Stress and Tissue Repair: Mechanism, Biomarkers, and Therapeutics

**DOI:** 10.1155/2021/6204096

**Published:** 2021-02-27

**Authors:** Reggiani Vilela Gonçalves, Andrea M. A. Costa, Lukasz Grzeskowiak

**Affiliations:** ^1^Animal Biology Department, Federal University of Viçosa, Viçosa, 36570-000 Minas Gerais, Brazil; ^2^Animal Biology and Cellular and Structural Biology Graduate Program, Federal University of Viçosa, Viçosa, 36570-000 Minas Gerais, Brazil; ^3^Morphological Science Graduate Program, State University of Rio de Janeiro, Rio de Janeiro 20950-003, Brazil; ^4^Institute of Animal Nutrition, Freie Universitat Berlin, Berlin 14195, Germany

During the “respiratory burst” in activated leukocytes, the release of reactive oxygen species (ROS) and reactive nitrogen species (RNS) occurs, and these phenomes present a central role in tissue repair [1, 2]. In the inflammatory phase, neutrophils reach the injured area, sending signals for the migration of macrophages that mediate the expression of cytokines, growth factors, reactive oxygen (ROS), and nitrogen species (RNS) [3]. These ROS and RNS generated during inflammation can lead to cell lesions such as membrane disorganization and protein oxidation by altering cellular functions. The balance between ROS production and antioxidant defense is important for efficient tissue repair in organs such as the skin, liver, lungs, kidneys, heart, and testes [4–6]. When the tissue is damaged by reactive species, it is common to observe lipid, protein, and DNA damage, leading to oxidative stress that disables tissue repair [5, 6].

During the chronification process, a persistent activation of the COX way occurs, and neutrophils and macrophages release cytokines and chemokines, which attract more cells to the location of the inflammation and promote oxidative stress in the repairing tissue [7, 8]. The excess of proinflammatory mediators promotes the increase of the peroxide of hydrogen (H_2_O_2_) and nitric oxide content, which accelerate the peroxidation of the component cells [7, 8]. Therefore, a controlled inflammation process is necessary to avoid persistent tissue damage through the continued action of free radicals and reactive oxygen species (ROS) [7]. Associated with this, we can highlight that the healing environment is usually prooxidant and generally presents a decrease in the synthesis and expression of antioxidant enzymes such as superoxide, glutathione, and catalase, impairing the healing environment [8, 9]. In general, a desirable repairing process results from the balance between the synthesis and degradation of inflammatory mediators and pro- and antioxidant compounds [9, 10].

Antioxidant systems seem to play a crucial role in maintaining the morphological and functional integrity of all microorganisms. Accordingly, disruptors of redox balance (e.g., inductors of oxidative stress or inhibitors of antioxidant molecules) have been proposed as candidates for new healing drugs. Ideally, modulators of redox systems should be able to influence fibroblast migration, proliferation, increased collagen synthesis, and scar tensile strength, a process partially mediated by ROS and RNS catalysis [11, 12]. Interestingly, several drugs and compounds developed for different conditions (e.g., NLRP3, propolis, carbon monoxide, *tea polysaccharides*, *safflor yellow* B, radiation, *Huangbai liniment*, lipoxin A4 ameliorates, resveratrol, and MiR-200c) exhibit antioxidant, anti-inflammatory, and antimicrobial properties. In many cases, these properties are based on the disruption of cell redox metabolism, a pharmacological effect that opens new venues for drug repurposing and the development of new strategies for the treatment of tissue injury.

This special issue gathers a set of 17 studies in an interdisciplinary platform that addresses the subcellular, cellular, and molecular bases of the metabolism redox associated with the recovery of damaged tissue. This special issue also highlights the continuing effort to understand the redox systems associated with repairing tissues in all levels and understand how the action of different treatments can lead to the increase of antioxidant enzyme levels, such as superoxide dismutase (SOD), catalase (CAT), and glutathione peroxidase (GPx), and consequently accelerate the healing process in different tissues. This issue contains seventeen papers describing different mechanisms involved in tissue repairing in different pathological conditions, which are briefly mentioned below.

Some studies have shown that external agents like alcohol, carbon monoxide, cadmium, and radiation exposition reflect an imbalance between oxidants and antioxidant mechanisms in favour of oxidants capable of provoking tissue damage. These prooxidant mechanisms are related to genetic and epigenetic regulation and can regulate the expression of molecules that can activate signal transduction pathways responsible for inflammation and cause oxidative damage to proteins, lipids, and DNA. The studies in this issue showed that the redox system begins producing free radicals a few hours after exposure and can upregulate several enzymes including nicotinamide adenine dinucleotide phosphate oxidase (NADPH oxidase), lipoxygenases (LOXs), nitric oxide synthase (NOS), and cyclooxygenase (COXs). These enzymes are expressed in specific ways in various cells, tissues, and organs. Normal cells that are exposed to these external agents will give rise to nuclear and mitochondrial DNA damage, which can lead to cell death via processes such as apoptosis and necrosis. Necrosis can trigger the release of inflammatory cytokines such as IL-1, IL-4, IL-13, and other inflammatory mediators, while apoptosis may cause the release of anti-inflammatory cytokines including TGF-*β* and IL-10.

Considering the development of new drugs and treatments, the studies published in this edition were very broad and addressed different types of therapies in the treatment of injuries in various systems. The use of natural products obtained from plants and animals were more common; among them, we can highlight propolis-nanofiber dressing used for burn treatment and the Huangbai liniment plant compost for accelerated wound healing by Nrf2 activation signalled in diabetes. In one study, Danhong injection was extracted from *Salviae Miltiorrhizae Radix* and *Carthami tinctorii Flos* and showed several positive effects, such as anti-inflammatory, antioxidant, and antiapoptotic, and prevented postoperative adhesion. In another study, resveratrol showed protection against thioacetamide toxicity in rat kidneys with respect to DNA damage, oxidative stress, renal function, and cytokine release.

In addition, two studies analysed the importance of nutrition for oxidative stress in tissues. The former study showed that nutrient intake and malnutrition can act as predictors of oxidative uremic toxicity. Therefore, they provided evidence that age and sex exhibited a limited association with malnutrition and cardiometabolic risk factors in haemodialysis patients. In addition, these authors showed that serum antioxidant mediators seem to have greater predictive sensitivity to estimate molecular oxidative damage in haemodialysis patients. The latter study evaluated glutamine supplementation for the treatment of acute pancreatitis and showed that the use of glutamine might be promising for the future management of acute pancreatitis by relieving the intracellular energy stress, thereby attenuating the predominance of necrosis over apoptosis. Moreover, one review summarized the role of macroautophagy in the heart following myocardial infarction and showed that some noncoding RNAs and their easy manipulation exposed their potential as new targets for clinical development to treat autophagy-related diseases. Identification of specific cardiac noncoding RNAs that regulate autophagy could be a good opportunity to protect the heart from myocardial infarction injury without affecting the autophagy activity in other organs.

We hope that the readers of this special issue will find these findings interesting and useful to advance the understanding of such a complex and multifaceted theme, suggesting an update for this interesting topic.
